# Prophylactic administration of metformin alleviates withdrawal symptoms associated with heroin

**DOI:** 10.3389/fphar.2025.1647624

**Published:** 2025-10-08

**Authors:** Minghong Liu, Junyu Jiang, Xiaolong Wu, Jiaxin Liu, Yang Liu, Shanshan Ling, Huichun Chen, Gongliang Zhang, Yuanhai Li, Gang Pang, Xinrong Tao

**Affiliations:** ^1^ Department of Anesthesiology, The First Affiliated Hospital of Anhui University of Science and Technology, Huainan, China; ^2^ Anhui University of Science and Technology, Huainan, China; ^3^ The First Affiliated Hospital of Anhui Medical University, Hefei, China; ^4^ Anhui Shendong Biotechnology Development Co., Ltd., Huainan, China; ^5^ College of Basic Medical Sciences, Anhui Medical University, Hefei, China

**Keywords:** metformin, withdrawal, hippocampus, behavior test, heroin, naloxone-induced

## Abstract

**Objective:**

This study aimed to evaluate whether metformin can alleviate heroin withdrawal symptoms and explore its underlying mechanisms, focusing on its reducing microglia-related neuroinflammation in the CA3 region of hippocampus.

**Methods:**

We set up a heroin withdrawal mouse model by the administration of naloxone in heroin-treated mice. To reduce experimental variables, only male mice were considered in this study. The behaviors of withdrawal model mice with saline, naloxone (5 mg/kg), or metformin (100 mg/kg) treatment (n = 12 for each group) were evaluated by Open Field Test (OFT) and Elevated Plus Maze Test. After the behavior tests, brain tissues were collected for histological staining experiments. Our study mainly focused on the hippocampal CA3 region. Protein expression levels of TLR4 (inflammation related) and BAX were analyzed using immunofluorescence staining.

**Results:**

Metformin was proved to be capable of improving movement-related and posture-related behavioral changes caused by the naloxone-induced heroin withdrawal. Furthermore, the results of the Open-Field Test and Elevated Plus Maze test were used to demonstrate metformin’s role in softening anxiety levels, which was due to its reducing microglia-related neuroinflammation in the CA3 region of hippocampus. This neuronal protection was achieved by downregulating the expression of TLR4 (inflammation related) and BAX (apoptosis marker) protein.

**Conclusion:**

Overall, our data suggests that prophylactic administration of metformin has a therapeutic effect on glial-induced neuroinflammation and neuronal apoptosis in heroin withdrawal mice. Histological experiments suggested that metformin reduced microglia-mediated neuroinflammation and downregulated TLR4 and BAX expressions in the hippocampus.

## Introduction

Heroin is a highly addictive illegal opioid derived from morphine. In recent years, the number of individuals dependent on heroin has been rising, particularly among adolescents (Kuehn, 2013; Dart et al., 2015; Griesler et al., 2019; Kelley-Quon et al., 2013). Heroin dependence is induced by activating opioid receptors (Le Roy et al., 2013), especially μ-opioid receptors (Kreek et al., 2012). The cycle of addiction is frequently sustained by the difficulty of managing withdrawal symptoms, which may include anxiety, irritability, muscle aches, and, in severe cases, seizures and psychosis (Verster et al., 2021). Withdrawal is widely recognized as a factor contributing to relapse, as well as to compulsive drug-seeking and drug-taking behaviors (Blum et al., 2013; Pang et al., 2016). Alongside physical symptoms, anxiety and depression are key emotional aspects of heroin withdrawal and are believed to drive continued use (Araujo et al., 1996).

Heroin withdrawal is characterized by a complex interplay of neurobiological changes, including neuroinflammation and neuronal apoptosis. Recent studies have shown that neuroinflammation, mediated by glial cells such as microglia, plays a crucial role in the development of withdrawal symptoms (Jacobsen et al., 2014; Parekh et al., 2020). Specifically, the activation of Toll-like receptor 4 (TLR4) in glial cells has been implicated in opioid-induced neuroinflammation, leading to increased expression of pro-inflammatory cytokines such as IL-6 (Toloff and Woodcock, 2022). This neuroinflammation is associated with increased anxiety and depressive behaviors, which are key emotional components of heroin withdrawal.

Metformin, a widely used antidiabetic medication, has recently been shown to have a range of biological functions beyond its anti-diabetic effects, including anti-inflammatory, anti-apoptotic, and neuroprotective properties (Alimoradi et al., 2021; Chomanicova et al., 2021; Varjabedian et al., 2018; Glossmann Hartmut and Lutz Oliver, 2019; Diaz et al., 2021). Recent evidence suggests that metformin may have therapeutic potential in the treatment of heroin withdrawal. For instance, metformin has been shown to reduce morphine tolerance by inhibiting microglial-mediated neuroinflammation (Pan et al., 2016). Furthermore, metformin’s ability to modulate the hypothalamic-pituitary-adrenal (HPA) axis, which is often dysregulated during withdrawal, may contribute to its efficacy in reducing withdrawal symptoms (Shin et al., 2021). Metformin also enhances the expression of brain-derived neurotrophic factor (BDNF), a neurotrophic factor that promotes neuronal resilience and may help alleviate withdrawal-induced anxiety and depression-like behaviors (Liu et al., 2023).

Despite these promising findings, the precise mechanisms by which metformin alleviates heroin withdrawal symptoms are not yet fully understood. The current study aims to investigate the effects of prophylactic metformin administration on withdrawal symptoms in a mouse model of heroin dependence, focusing on its potential to reduce microglia-related neuroinflammation and neuronal apoptosis in the hippocampal CA3 region. We hypothesize that metformin’s therapeutic effects are mediated by its ability to downregulate TLR4 expression and reduce BAX (apoptosis marker) levels, thereby alleviating withdrawal symptoms and improving behavioral outcomes.

## Materials and methods

### Animals

Adult 8 to 12-week-old male C57BL/6 mice weighing 22 ± 2 g were obtained from the Experimental Animal Center of Anhui Medical University (Hefei, China) and housed in groups of four per standard polycarbonate cage (30 cm × 18 cm x 12 cm) with *ad libitum* access to food and water in a controlled environment. All procedures were conducted by following the guidelines as described in the NIH Guide for the Care and Use of experimental animals and were approved by Anhui Medical University Biomedical Ethics Committee.

### Heroin withdrawal mouse model

Mice received subcutaneous heroin twice daily starting at a dose of 10 mg/kg and the dose was increased by 5 mg/kg each time until reaching 50 mg/kg by the fifth day (Wu et al., 2015; Li et al., 2022). On day 5, mice received 50 mg/kg heroin treatment at 8:00 AM. Naloxone was administered 2 h later to precipitate jumping and other withdrawal symptoms (El-Kadi and Sharif, 1995). Behavioral assessment was conducted with a Noldus PhenoTyper system. Mice were individually placed into clear square observation chambers (30 cm by 30 cm by 35 cm tall) with bedding at the bottom. The heroin withdrawal behaviors included jumping (all feet off the floor), body grooming, rearing, wet dog shakes (whole body shakes), paw licking, and extended posture. Each behavior was counted for 30 min immediately after the naloxone challenge. The distance moved and immobility in heroin-withdrawal mice treated with saline/metformin were also recorded for 60 min beginning immediately after naloxone injection.

### Compounds

Heroin was obtained from the Department of Public Security of Anhui Province (Hefei, China). Metformin (HY-B0627) was purchased from MedChemExpress Co. Ltd (Hefei, China). Naloxone was purchased from Kangze Pharmaceutical Co. Ltd (Hunan, China). All chemicals were dissolved in sterile 0.9% saline solution. Naloxone was administered at 5 mg/kg (Li et al., 2022; Shah et al., 2008). In the early stage, we conducted dose tests for metformin at 50 mg/kg, 100 mg/kg and 200 mg/kg to choose the best dose (100 mg/kg) (See Supplementary Figure S1 in Supplementary Data). To be noticed, metformin should be administered 30 min before each heroin injection as a pretreatment.

### Study design

Mice were randomly assigned into 3 groups (*n* = 12 for each group). The study design for each group was shown in Table 1. The detailed administration strategy for each group per day was shown in Table 2.

**TABLE 1:** Study design.

Group	Treatment	Mice number	Behavioral test
Saline	naloxone (5 mg/kg) saline	12	posture-related behavioral records (30 min); movement-related behavioral records in OFT (60 min); EPM (5 min); Marble burying test (30 min)
Heroin	naloxone (5 mg/kg) heroin (10–50 mg/kg)	12	posture-related behavioral records (30 min); movement-related behavioral records in OFT (60 min); EPM (5 min); Marble burying test (30 min)
Met + heroin	naloxone (5 mg/kg) heroin (10–50 mg/kg) metformin (100 mg/kg)	12	posture-related behavioral records (30 min); movement-related behavioral records in OFT (60 min); EPM (5 min); Marble burying test (30 min)

**TABLE 2:** Administration strategy.

Group	Day 1	Day 2	Day 3	Day 4	Day 5
Saline	8:00 a.m.: saline 8:00 p.m.: saline	8:00 a.m.: saline 8:00 p.m.: saline	8:00 a.m.: saline 8:00 p.m.: saline	8:00 a.m.: saline 8:00 p.m.: saline	8:00 a.m.: saline; 10:00 a.m.: naloxone (5 mg/kg)
Heroin	8:00 a.m.: heroin (10 mg/kg) 8:00 p.m.: heroin (15 mg/kg)	8:00 a.m.: heroin (20 mg/kg) 8:00 p.m.: heroin (25 mg/kg)	8:00 a.m.: heroin (30 mg/kg) 8:00 p.m.: heroin (35 mg/kg)	8:00 a.m.: heroin (40 mg/kg) 8:00 p.m.: heroin (45 mg/kg)	8:00 a.m.: heroin (50 mg/kg); 10:00 a.m.: naloxone (5 mg/kg)
Met + heroin	7:00 a.m.: metformin (100 mg/kg) 8:00 a.m.: heroin (10 mg/kg) 8:00 p.m.: heroin (15 mg/kg)	7:00 a.m.: metformin (100 mg/kg) 8:00 a.m.: heroin (20 mg/kg) 8:00 p.m.: heroin (25 mg/kg)	7:00 a.m.: metformin (100 mg/kg) 8:00 a.m.: heroin (30 mg/kg) 8:00 p.m.: heroin (35 mg/kg)	7:00 a.m.: metformin (100 mg/kg) 8:00 a.m.: heroin (40 mg/kg) 8:00 p.m.: heroin (45 mg/kg)	7:00 a.m.: metformin (100 mg/kg) 8:00 a.m.: heroin (50 mg/kg); 10:00 a.m.: naloxone (5 mg/kg)

Behavioral tests were performed immediately after the naloxone injection in a sequential order: posture-related behavioral records (30 min); movement-related behavioral records in OFT (60 min); EPM (5 min); Marble burying test (30 min). All behavioral tests were evaluated by recorded videos, which would result in some behavioral indicators of some mice being not accurately recorded. Behavioral indicators of each mouse, especially those posture-related [including jumping (all feet off the floor), body grooming, rearing, wet dog shakes (whole body shakes), paw licking, and extended posture], were counted by at least three researchers. A mouse was excluded for an indicator statistical analysis if its records of the indicator were inconsistent among researchers.

## Behavioral test

### Basal locomotor activity recording with an open field test (OFT)

The mouse was placed in the center of a white opaque arena (30 cm by 30 cm by 37.5 cm tall) and tracked via an overhead video camera interfaced with behavioral tracking software EthoVision XT 5.1 (Noldus Information Technology, Netherlands). The recorded movement of the mice’s center point in centimeter throughout for the trial [distance moved (cm)] and the percentage of time that EthoVision failed to detect any linear or angular movement of the mouse [immobility (%)] were calculated.

### Elevated plus maze test

After the OFT, mice were placed in the center area of the elevated plus maze and faced the open arm. The behaviors were detected via an overhead video camera fixed to the roof for 5 min for analyzing the number of entering the open arms; and percentage of time spent in open arms, which analyzed by behavioral tracking software EthoVision XT 5.1 (Noldus Information Technology, Netherlands).

### Marble burying test

All tests were carried out between 19:00 and 22:00, in a quiet environment with a stable temperature (22 °C–24 °C). Mice were habituated to the experimenting room for 30 min before being placed into a polypropylene cage (30 cm × 18 cm x 12 cm) containing 20 glass marbles (1.5 cm diameter) evenly spaced on 5 cm deep rodent bedding. After 30 min of observation, if three-fourths of the surface area of the glass marbles is covered by the rodent bedding, the score will be assigned as buried marble.

## Histological staining

### Brain tissue acquisition and processing

After the behavioral test, mice were deeply anesthetized with 3% chloral hydrate, open the thoracic cavity and expose heart, then perfused transcardially with 4 °C phosphate-buffered saline (PBS) and 4% paraformaldehyde (PFA) in 0.1 M PBS. After cervical dislocation resulting in euthanasia, brain tissue was carefully separated and immersed in 4% PFA for the following histological experiment. To be noticed, only three mice’s brain tissue in each group were randomly selected for the histological analysis.

### Immunohistochemistry staining

Dissected brain tissue was fixed in 4% PFA for 24–48 h, then processed for paraffin-embedding and sectioning. In this experiment, we made 4 μm-thickness coronal hippocampus slices. Briefly, the slices were baked in 60 °C for 8 h, and deparaffined in xylene for 30 min, hydrated in gradient alcohol, then rinsed in dd-H_2_O for 5 min. Next, we processed the Antigen retrieval with EDTA (pH = 8.0) (Servicebio, G1206) for 30 min via steam. Endogenous peroxidase was blocked by 3% H_2_O_2_ for 15 min, 5% BSA mixed with 0.3% Triton X-100 for tissue block and tissue permeability for 1 h. Primary antibody was incubated at 4 °C overnight. The secondary antibody, which mentioned above, was incubated after rewarming for 1 h at room temperature. Finally, DAB color-substrate kit (Vector Laboratories, SK-4100) was applied for 10–20 min, and terminal the chromogenic reaction by dd-H_2_O. Images were obtained with BX50 microscope (Olympus, Tokyo, Japan).

### Immunofluorescence staining

The process of the hippocampus section was mentioned above. Before embedding, the brain tissue was soaked in 30% sucrose solution. Coronal, 5 μm thickness sections were obtained. Baked, hydrated and deparaffined as discussed above. Then EDTA (pH = 8.0) (Servicebio, G1206) were used for antigen retrieval via microwave for 15 min. After natural cooling, the slices were shaken on the decolorizing shaker 3 times and 5 min each, 5% BSA block for 30 min. Next, primary antibodies were incubated in 4 °C overnight in the dark. After rewarming and washing, secondary antibodies, which mentioned above, were incubated at 1:1,000 dilution ratio for 50 min in the dark. Nuvleus were localized with DAPI (Servicebio, G1012) for 10 min at room temperature in the dark. The images were detected by Nikon Eclipse C1 confocal microscope (Nikon, Japan). And quantified using ImageJ software.

### Statistical analysis

Data were expressed as mean ± S.E.M (with error bars). Statistical differences in distance travel and immobility in 5-min bins were analyzed with a two-way repeat measures ANOVA. Student’s t-test or one-way ANOVA were also used. If significance was found, post-hoc Bonferroni or Dunnett’s multiple comparison was used. *P* < 0.05 was considered statistically significant.

## Results

### Metformin changes the movement distance and the immobility percentage of mice undergoing naloxone-precipitated heroin withdrawal

**FIGURE 1:**
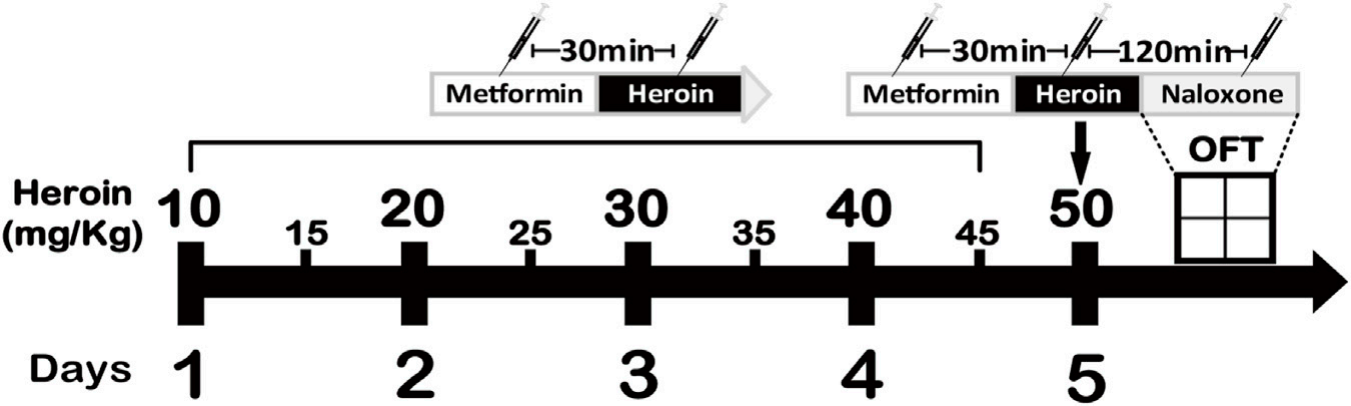
The experimental procedure. Induction of heroin dependency by administering increasing doses of heroin is shown in the graph.

**FIGURE 2:**
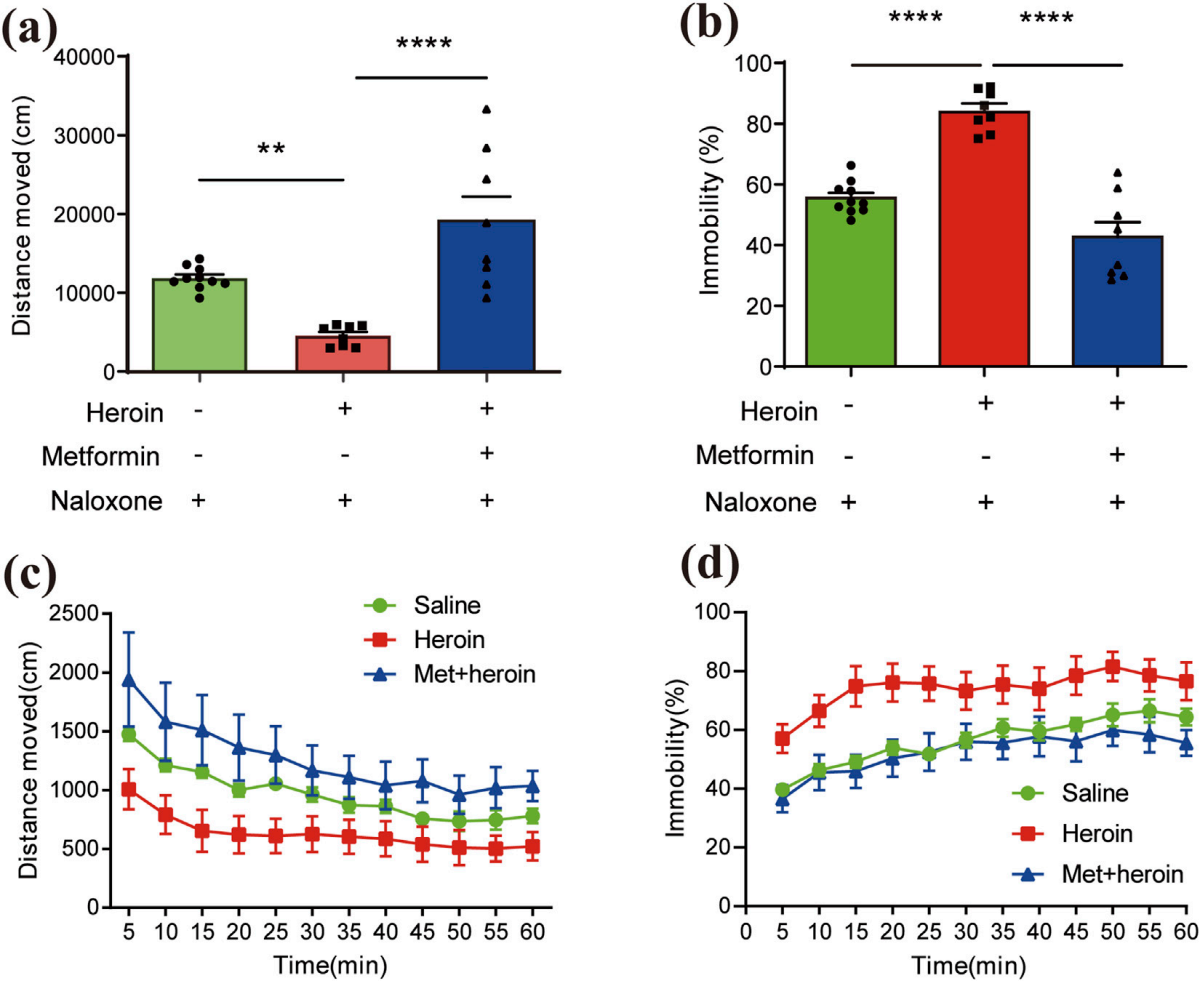
Features of behavioral symptoms induced by acute heroin withdrawal in mice. Bar plots of **(a)** distance moved (cm) and **(b)** immobility (%) within an hour. Pillars colored by different administration groups. Line charts of **(c)** distance moved (cm) records and **(d)** immobility (%) across 1 hour. Lines colored by different administration groups. Data are expressed as mean ± SEM; N = 8–12 for each group. One-way/two-way ANOVA was used to analyze significant differences between groups. *P < 0.05; **P < 0.01; ***P < 0.001.

Heroin addictive murine model was established as illustrated in Figure 1. Heroin dependence was induced with a 5-day regimen of escalating heroin doses, followed by metformin administration for treatment. Metformin significantly increased distance traveled ( F (Dart et al., 2015; Li et al., 2022) = 17.34, *P* < 0.0001, Figure 2a) and reduced immobility ( F (Dart et al., 2015; Li et al., 2022) = 44.00, *P* < 0.0001, Figure 2b) within 1 hour, as demonstrated by one-way ANOVA and post-hoc Dunnett’s multiple comparisons, compared to saline treatment. A two-way repeated measures ANOVA on distance moved in 5-min intervals showed a significant main effect of treatment ( F (11, 384) = 4.689, *P* < 0.0001) and of time bin ( F (2, 384) = 40.05, *P* < 0.0001), with no significant treatment × time bin interaction ( F (22, 384) = 0.2531, *P* = 0.9998; Figure 2c). Similarly, immobility measured in 5-min intervals showed a significant treatment effect ( F (11, 384) = 5.228, *P* < 0.0001) and time-bin effect ( F (2, 384) = 58.71, *P* < 0.0001), with the control treatment showing no significant time-box interaction effect ( F (22, 384) = 0.2411, *P* = 0.9999; Figure 2d).

### Metformin alleviates withdrawal symptoms precipitated by naloxone in heroin-treated mice

**FIGURE 3:**
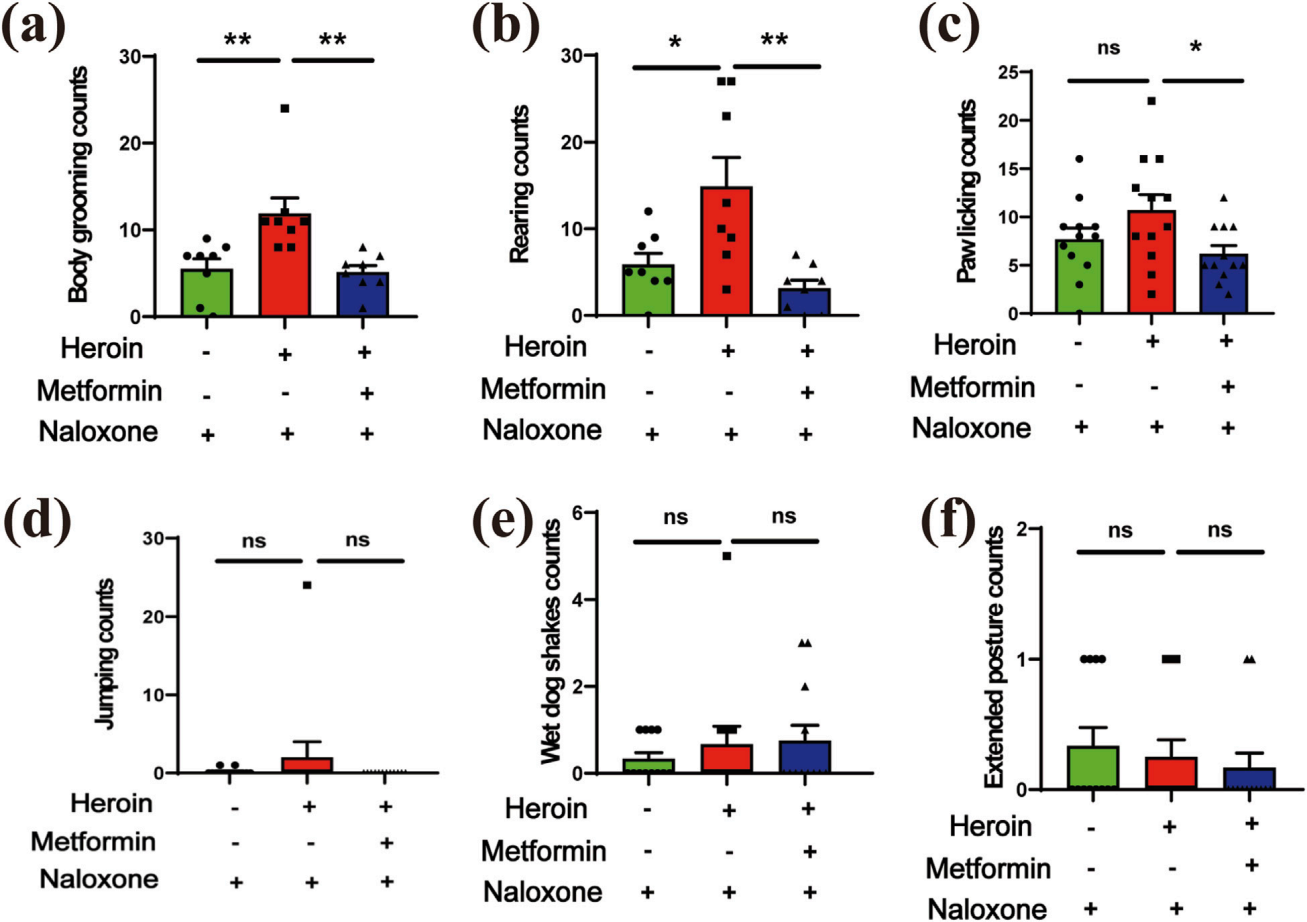
The withdrawal symptoms. **(a)** body grooming (counts). **(b)** rearing (counts). **(c)** paw licking (counts). **(d)** jumping (counts). **(e)** wet dog shakes (counts). **(f)** extended posture. Data are shown as mean ± SEM from at least three independent experiments; one-way ANOVA was used to analyze significant differences between groups. Sample size N = 8–12. *P < 0.05; **P < 0.01; ***P < 0.001.

To further test if metformin could alleviate naloxone-induced withdrawal symptoms in heroin-treated mice or not, the withdrawal behaviors in each group were recorded over a 30-min period. The observed behaviors included body grooming, rearing, paw licking, jumping, wet dog shakes, and extended posture. Metformin-treated mice displayed reduced activity levels in these experiments. In heroin-withdrawn mice, metformin treatment significantly reduced body grooming (Figure 3a), rearing (Figure 3b), and paw licking (Figure 3c) (P < 0.05). Additionally, metformin showed a non-significant trend toward reducing jumping (Figure 3d), wet dog shakes (Figure 3e), and extended posture (Figure 3f) in these mice.

### Metformin alleviates anxiety behaviors induced by heroin withdrawal in heroin-treated mice

**FIGURE 4:**
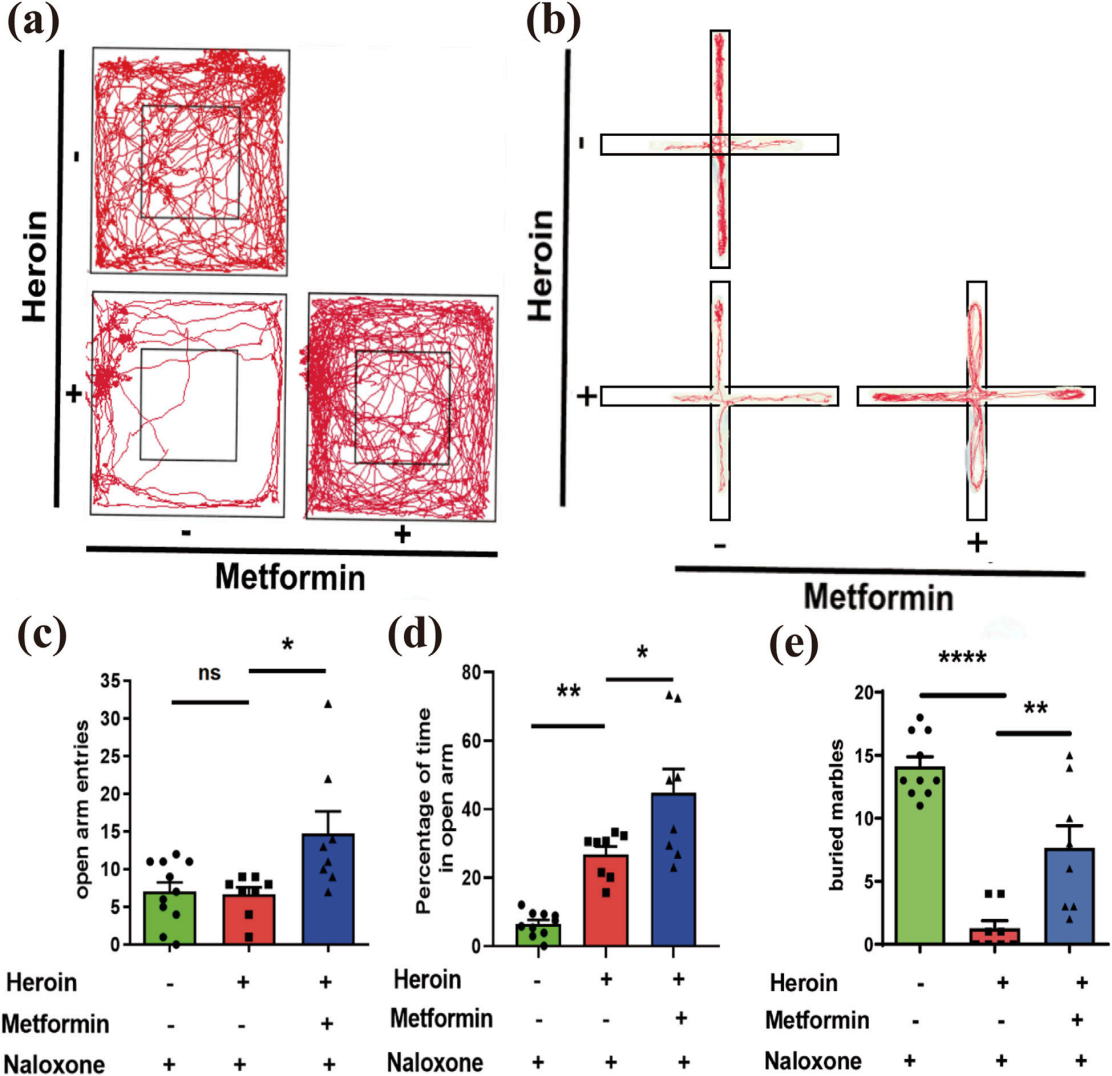
Behavioral Experiments. **(a)** Representative movement track pictures of mice in OFT. **(b)** Representative movement track diagrams of mice in EPM. **(c)** number of entries into the open arms in EPM. **(d)** Percentage of time in the open arms in EPM. **(e)** Marble burying. Mean ± SEM of the marbles buried by the mice after 30 min (N = 8–12 in each group). Data are shown as mean ± SEM from at least three independent experiments; one-way ANOVA was used to analyze significant differences between groups. *P < 0.05; **P < 0.01; ***P < 0.001.

The Open Field Test (OFT) revealed that time spent in the center arena decreased following heroin withdrawal, while metformin treatment significantly increased this time ( F (Dart et al., 2015; Li et al., 2022) = 11.69, *P* = 0.0003, one-way ANOVA with post-hoc Dunnett’s multiple comparisons; Figure 4a). Following the OFT, we conducted the Elevated Plus Maze (EPM) test. One-way ANOVA with post-hoc Dunnett’s comparisons indicated that metformin treatment significantly increased the number of open-arm entries ( F (Dart et al., 2015; El-Kadi and Sharif, 1995) = 5.933, *P* = 0.0081; Figure 4b) and the percentage of time spent in the open arms ( F (Dart et al., 2015; Li et al., 2022) = 23.56, *P* < 0.0001; Figures 4c,d). Additionally, the marble burying test showed that metformin-treated mice buried more marbles, which is associated with reduced anxiety behavior, compared to the withdrawal-only group ( F (Dart et al., 2015; Li et al., 2022) = 33.34, *P* < 0.0001; Figure 4e).

### Metformin reduces microglial-induced neuroinflammation in the hippocampal CA3 region

**FIGURE 5:**
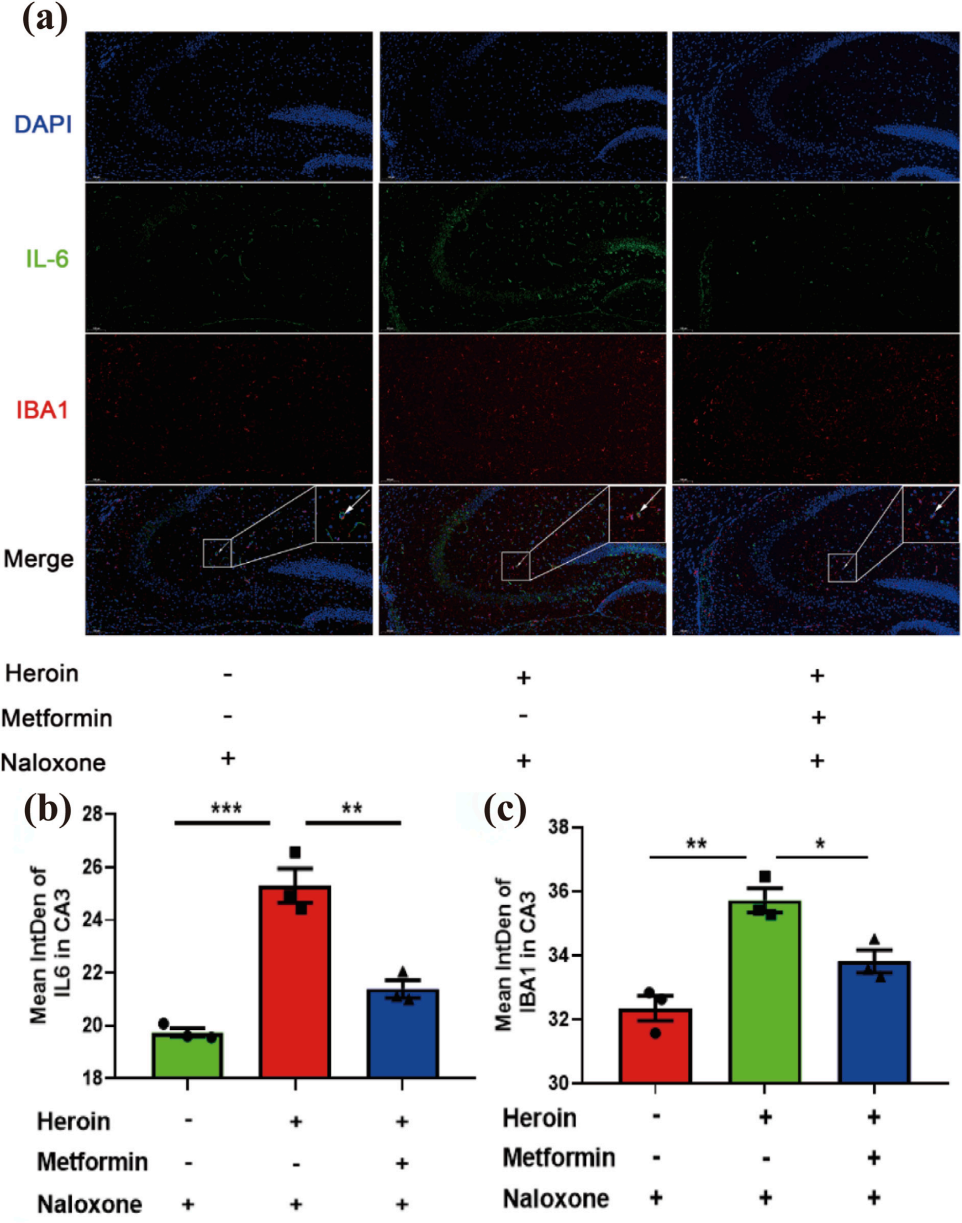
Immunofluorescence double-labeling staining analysis of IL-6 and IBA1 co-expression in hippocampus CA3. **(a)** Distribution of IL-6 and IBA1 in hippocampus CA3 Double immunofluorescence of IBA1 (red)/IL-6 (green) labeled nucleus of cells. Nuclei were stained with DAPI (blue). **(b)** Bar graphs show semi-quantitative evaluation of IL-6 co-expression immunofluorescence in CA3. **(c)** Bar graphs show semi-quantitative evaluation of IBA1 co-expression immunofluorescence in CA3. Scale bar 100 μm. Data are shown as mean ± SEM from at least three independent experiments; one-way ANOVA was used to analyze significant differences between groups. N = 3 in each group. *P < 0.05; **P < 0.01; ***P < 0.001.

Previous studies have shown that opioids can stimulate microglia-induced neuroinflammation *in vitro*. To examine whether neuroinflammation plays a role in acute heroin withdrawal, we performed double-stained immunofluorescence analysis. Our findings indicate that heroin withdrawal activates microglia and increases the expression of the pro-inflammatory cytokine interleukin-6 (IL-6). Additionally, our experiments demonstrated that the microglial marker ionized calcium-binding adapter molecule 1 (IBA1) colocalizes with IL-6, particularly in the hippocampal CA3 region (Figure 5a). Heroin withdrawal significantly upregulated the expression of IL-6 and Iba1 in the CA3 region of the hippocampus (Figures 5b,c).

### Metformin reduces TLR4 expression in glial cells within the hippocampus

**FIGURE 6:**
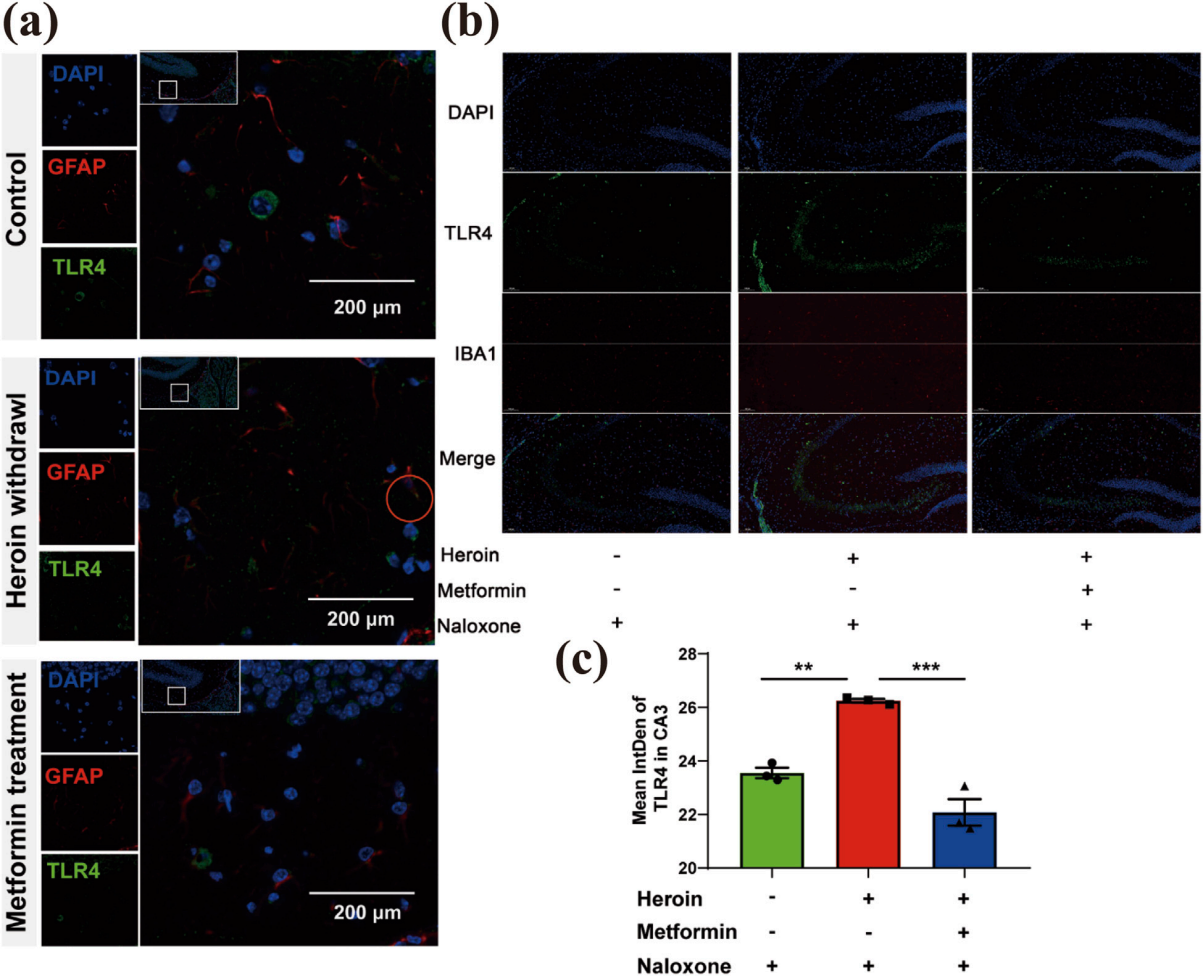
Basal locomotor activity recording with an Open field test. **(a)** Distribution of GFAP and TLR4 in glial cells (provide support neuron) Double immunofluorescence of GFAP (red)/TLR4 (green) labeled nucleus of cells. The red circle indicates three colors colocalization. **(b)** Distribution of IBA1and TLR4 in glial cells (provide support neuron) Double immunofluorescence of IBA1 (red)/TLR4 (green) labeled nucleus of cells. **(c)** Bar graphs show Mean intDen of TLR4 in CA3. Data are shown as mean ± SEM from at least three independent experiments; one-way ANOVA was used to analyze significant differences between groups. N = 3 in each group. *P < 0.05; **P < 0.01; ***P < 0.001.

Recently, TLR4’s role in opioid addiction has attracted significant interest. In our study, heroin withdrawal led to an upregulation of TLR4 expression in immunofluorescence staining, suggesting that the innate immune system is involved in heroin withdrawal. TLR4 was found to be primarily distributed in glial cells. Using immunofluorescence, we colocalized TLR4 with the astrocyte marker GFAP and the microglial marker IBA1. Notably, TLR4 colocalized with GFAP but not IBA1 (Figures 6a,b), indicating that astrocytes contribute to heroin withdrawal. Prophylactic administration of metformin significantly suppressed TLR4 expression in the CA3 region (Figure 6c).

### Metformin restored BAX expression to normal level following heroin withdrawal in hippocampal neurons

**FIGURE 7:**
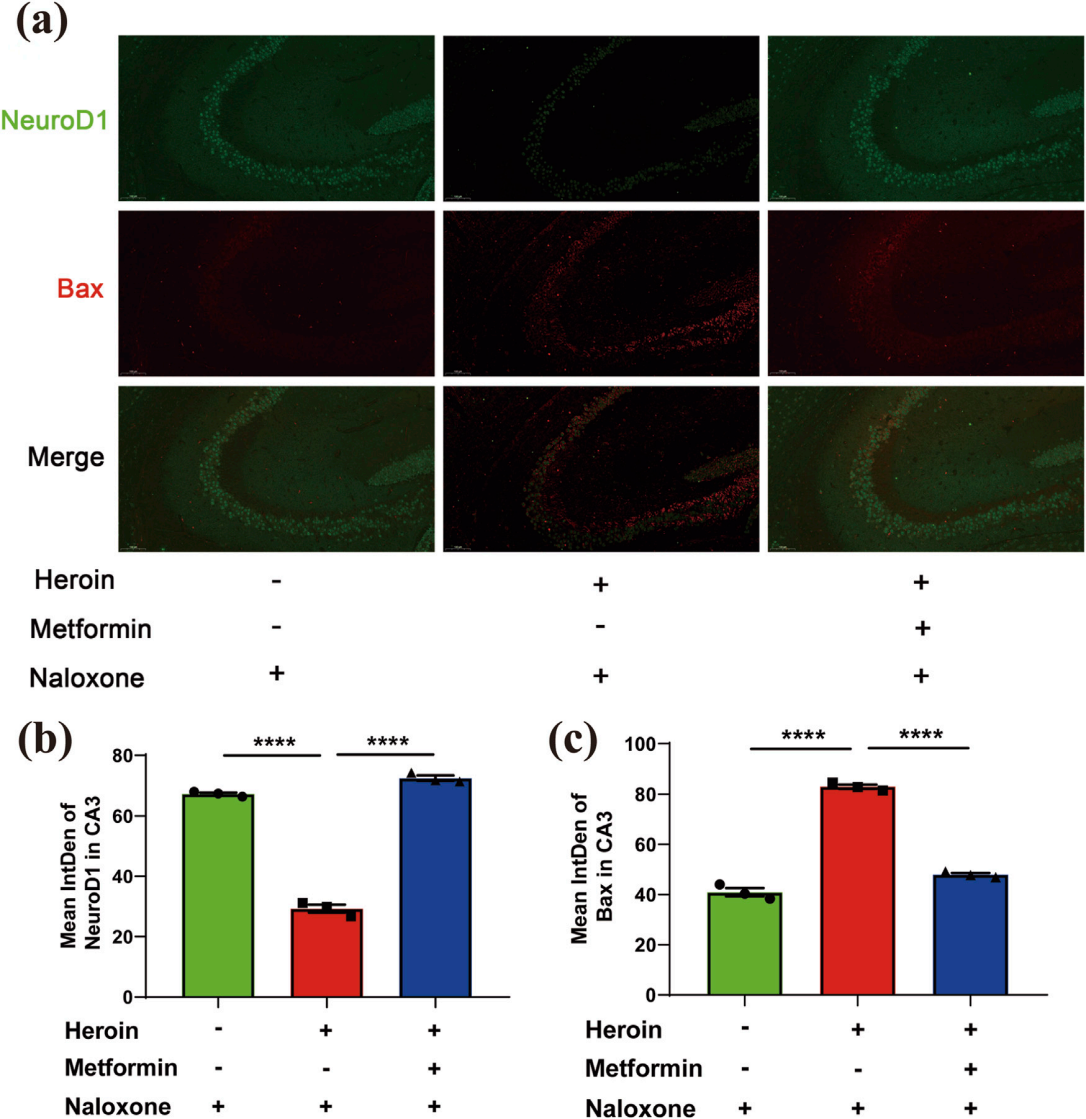
Immunofluorescence double-labeling staining analysis of NeuroD1 and Bax co-expression in hippocampus. **(a)** Representative immunofluorescence images of NeuroD1 and Bax in hippocampus DG and CA1. **(b)** Mean intden of NeuroD1 and Bax in DG/CA1. **(c)** Representative immunofluorescence images of NeuroD1and Bax in hippocampus CA3. Data are shown as mean ± SEM from at least three independent experiments; one-way ANOVA was used to analyze significant differences between groups. N = 3 in each group. *P < 0.05; **P < 0.01; ***P < 0.001.

Previous studies have shown that opioid withdrawal can lead to various forms of brain injury, affecting regions such as the nucleus accumbens (NAc) and the ventral tegmental area (VTA). However, damage to the hippocampus has rarely been addressed. Results showed that the expression of NeuroD1, a transcription factor crucial for development and function in the nervous system, decreased and the apoptosis marker BAX increased following heroin withdrawal. Prophylactic metformin administration before heroin exposure significantly restored both NeuroD1 and BAX levels to normal in the hippocampus, as shown in quantitative analyses (Figure 7). These results indicated that met.

## Discussion

Heroin withdrawal results in numerous behavioral changes, including anxiety and depressive behaviors (Ye et al., 2018; Ali et al., 2003; Leach et al., 2012; Latif et al., 2019; Lozano-López et al., 2021). Additionally, heroin withdrawal induces molecular changes in specific brain regions and neurotransmitters, particularly increasing levels of pro-inflammatory cytokines (Jacobsen et al., 2014; Parekh et al., 2020). Metformin, a classic hypoglycemic drug, has recently been reported to have various pharmacologic actions beyond its anti-hyperglycemic effects, including anti-inflammation, anti-aging, anti-apoptosis, and antineoplastic (Lv and Guo, 2020; Drzewoski and Hanefeld, 2021). Several studies have explored its treatments for drugs withdrawal that opioid receptors (Pan et al., 2016; Brynildsen et al., 2018). However, these adjunct therapies have shown limited success and are often associated with adverse side effects. Further research is needed to mitigate the negative effects of heroin withdrawal symptoms by targeting inflammation and metabolic pathways (Zhang et al., 2021).

With both movement-related and posture-related behavioral experiments, metformin was shown to alleviate heroin withdrawal symptoms in our study. Mice treated with metformin during detoxification were more likely to move positively and exhibited less grooming/jumping counts, which indicated that metformin was able to reduce the anxiety effects of heroin withdrawal. These results were further verified by two classic murine anxiety-like behaviors assessment methods (the Open-Field Test and Elevated Plus Maze) in this study (Prut and Belzung, 2003; Walf and Frye, 2007). The findings suggest that metformin effectively alleviates anxiety-like behaviors associated with heroin withdrawal.

The crosstalk between glial cells and neurons is a critical mechanism underlying the behavioral and molecular changes observed during heroin withdrawal (Saba, 2023; Tizabi et al., 2024). Numerous studies have found that glial cells, including astrocytes, microglia, and oligodendrocytes, affect neuronal function and synaptic plasticity by participating in the regulation of ion balance, neurotransmitter clearance, and metabolism, thereby affecting the occurrence and severity of withdrawal symptoms (Khakh et al., 2017; Shin et al., 2024). Microglia, the resident immune cells of the central nervous system, play a pivotal role in mediating neuroinflammation during withdrawal. Activated microglia releases pro-inflammatory cytokines such as IL-6, which can modulate neuronal activity and contribute to withdrawal symptoms (Jacobsen et al., 2014). Our study demonstrated that heroin withdrawal significantly upregulated the expression of IL-6 and the microglial marker IBA1 in the hippocampal CA3 region, with these markers colocalizing, indicating active glial-neuron interactions (Parekh et al., 2020).

Metformin’s ability to reduce microglial activation and subsequent neuroinflammation is likely a key mechanism by which it alleviates withdrawal symptoms. By downregulating TLR4 expression in glial cells, metformin may inhibit the activation of downstream inflammatory pathways, thereby reducing the release of pro-inflammatory cytokines and mitigating neuroinflammation (Pang et al., 2016). This reduction in neuroinflammation likely contributes to the observed behavioral improvements, as inflammation is known to exacerbate anxiety and depressive behaviors (Navarrete et al., 2022).

Moreover, the reduction of BAX expression in hippocampal neurons following metformin treatment suggests that metformin may also protect neurons from apoptosis. The hippocampus is particularly vulnerable to drug-induced neurotoxicity, and the preservation of neuronal integrity in this region is crucial for maintaining cognitive and emotional function (Albadawi, 2025). By reducing apoptosis and neuroinflammation, metformin may enhance neuronal resilience and promote recovery during withdrawal.

While our study provides valuable insights into the therapeutic potential of metformin in alleviating heroin withdrawal symptoms, it is essential to acknowledge several limitations and outline future directions for more comprehensive research.

Firstly, our study focused on male mice only, which is a significant limitation given that sex differences in opioid withdrawal and response to treatments have been documented in previous research. Studies have shown that female mice may exhibit more severe withdrawal symptoms compared to males, potentially due to differences in hormonal profiles and neurobiological responses to opioids (Bobzean et al., 2019; Townsend et al., 2021; Downs et al., 2024). For instance, estrogen levels have been shown to influence opioid sensitivity and withdrawal severity (Ethridge and Smith, 2023). Additionally, there is evidence suggesting that females may have a different response to metformin compared to males, which could impact the therapeutic outcomes (Ilias et al., 2022). Future studies should include both male and female mice to fully understand the sex-specific effects of metformin on heroin withdrawal.

Secondly, while our immunofluorescence findings suggest changes in TLR4 and BAX expression, these results should be validated using additional quantitative methods such as Western blot or ELISA. This would provide a more robust and comprehensive understanding of the molecular changes occurring in the hippocampus during heroin withdrawal and the effects of metformin treatment.

Thirdly, the inclusion of a selective TLR4 antagonist treatment group alongside metformin would strengthen the mechanistic link between TLR4 downregulation and the observed behavioral improvements. This would help confirm that the therapeutic effects of metformin are indeed mediated through its impact on TLR4 signaling pathways.

Moreover, future research should explore the long-term effects of metformin treatment on heroin withdrawal, including potential changes in cognitive function, long-term neuroinflammation, and neuronal resilience. Longitudinal studies could provide valuable insights into the sustained efficacy of metformin and its potential role in preventing relapses.

Additionally, further investigation into the role of other neuroinflammatory markers and pathways beyond TLR4 and BAX is warranted. This could include examining the expression of other cytokines, chemokines, and signaling molecules involved in the neuroinflammatory response to heroin withdrawal.

Finally, translational studies in humans are needed to validate the findings from animal models. Clinical trials assessing the safety and efficacy of metformin in treating heroin withdrawal symptoms in humans would be a crucial next step in translating these findings into practical therapeutic applications.

## Conclusion

Collectively, metformin significantly alleviated withdrawal symptoms in mice undergoing naloxone-induced heroin withdrawal. Prophylactic administration of metformin also reduced anxiety-like behaviors associated with heroin withdrawal. Furthermore, metformin inhibited neuroinflammation triggered by microglial cells in the CA3 region and reduced TLR4 expression in hippocampal glial cells. Heroin withdrawal was associated with an increase in the apoptotic marker Bax within hippocampal neurons, and preemptive metformin treatment effectively restored Bax levels to baseline. Further research is needed to elucidate the underlying mechanisms, particularly the intricate crosstalk between glial cells and neurons that mediates the therapeutic effects of metformin in heroin withdrawal.

## Data Availability

The datasets presented in this study can be found in online repositories. The names of the repository/repositories and accession number(s) can be found in the article/Supplementary Material.
