# Supplementary figures and images for: Prophylactic administration of metformin alleviates withdrawal symptoms associated with heroin
Source: Front Pharmacol. 2025 Oct 8;16:1647624. doi: 10.3389/fphar.2025.1647624 (PMC12541182; doi:10.3389/fphar.2025.1647624)

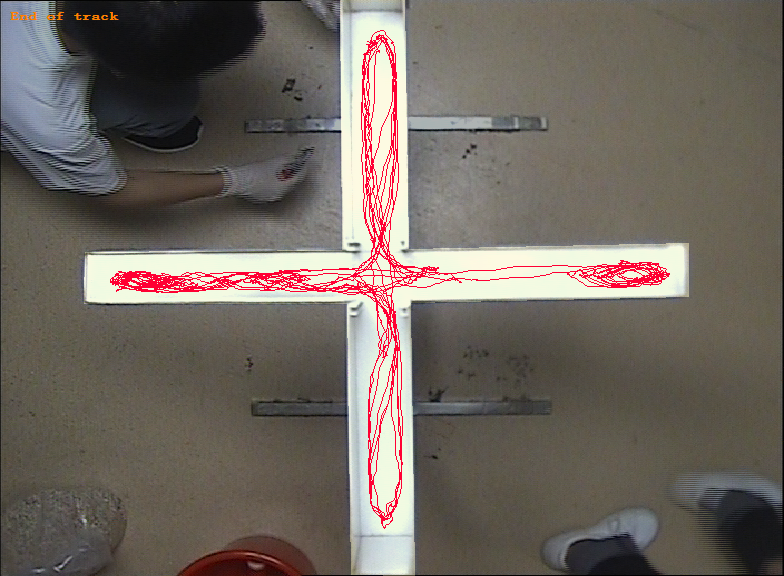

Supplement: Supplementary file 1 [file DataSheet1.zip › Supplementary Data/EPM/Heroin+Met-5.png]

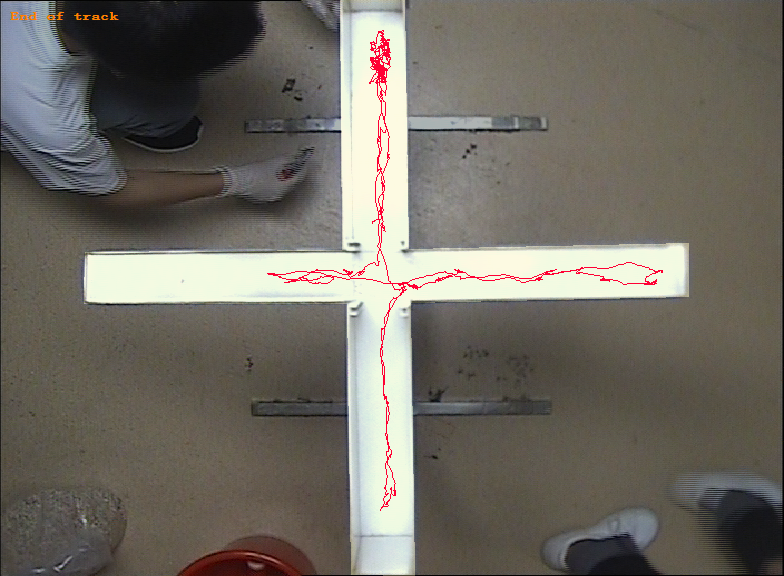

Supplement: Supplementary file 1 [file DataSheet1.zip › Supplementary Data/EPM/Heroin-11.png]

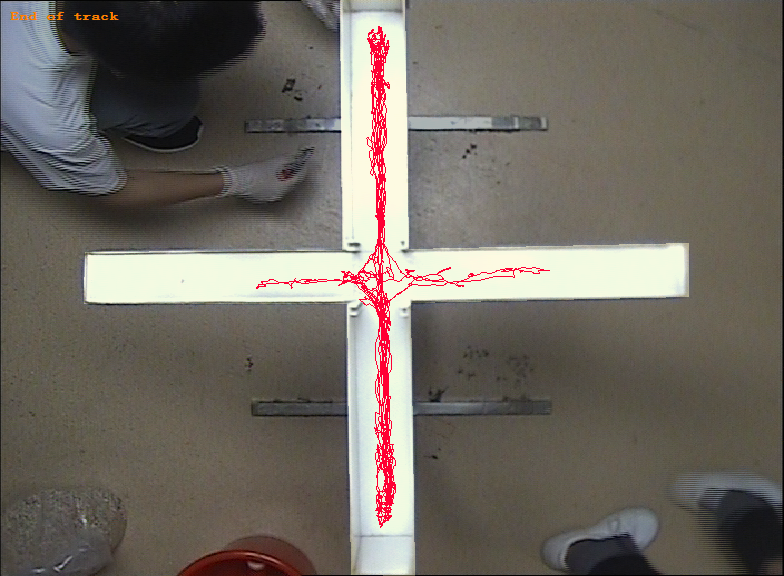

Supplement: Supplementary file 1 [file DataSheet1.zip › Supplementary Data/EPM/Saline-6.png]

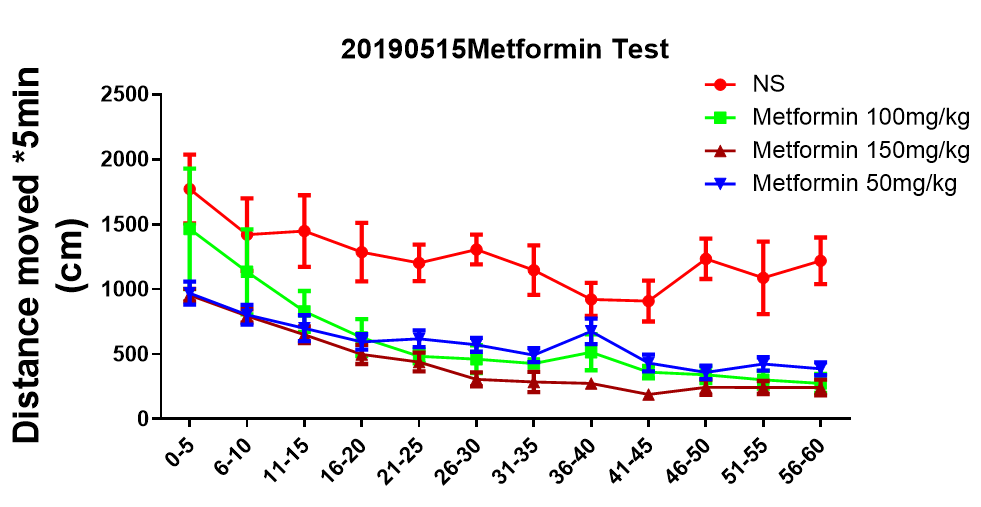

Supplement: Supplementary file 1 [file DataSheet1.zip › Supplementary Data/Fig S1.tif]

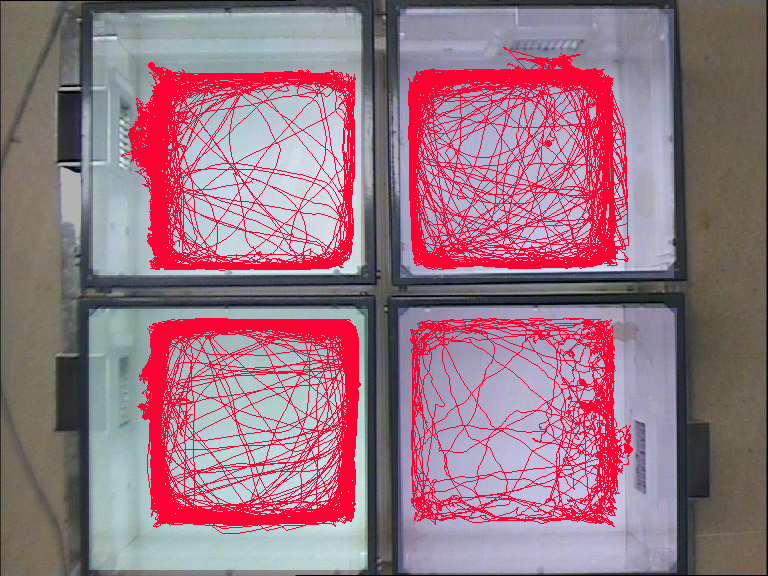

Supplement: Supplementary file 1 [file DataSheet1.zip › Supplementary Data/OF/Heroin+Met-2.png]

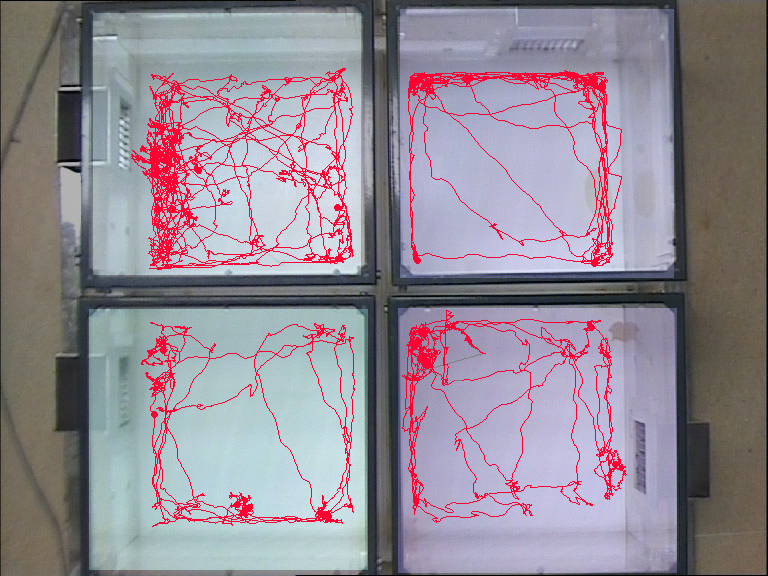

Supplement: Supplementary file 1 [file DataSheet1.zip › Supplementary Data/OF/Heroin-2.png]

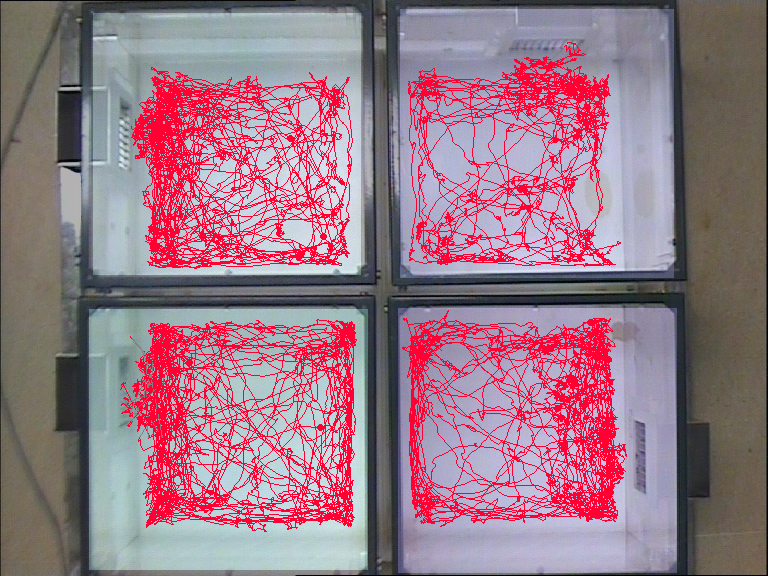

Supplement: Supplementary file 1 [file DataSheet1.zip › Supplementary Data/OF/Saline-3.png]
